# Implementing a colostrum-kit reduces the time to first colostrum for neonates admitted to the NICU – a retrospective observational study

**DOI:** 10.1186/s13006-024-00682-5

**Published:** 2024-11-15

**Authors:** Sara Hellström, Karolina Linden, Verena Sengpiel, Anders Elfvin

**Affiliations:** 1 Department of Paediatrics, Region Västra Götaland, The Queen Silvia Children’s Hospital, Sahlgrenska University Hospital, Gothenburg, Sweden; 2 Department of Paediatrics, Institute of Clinical Sciences, University of Gothenburg Sahlgrenska Academy, Gothenburg, Sweden; 3 Institute of Health and Care Sciences, Sahlgrenska Academy, University of Gothenburg, Gothenburg, Sweden; 4 Centre of Perinatal Medicine and Health, Institute of Clinical Sciences, Sahlgrenska Academy, University of Gothenburg, Gothenburg, Sweden; 5 Department of Obstetrics and Gynaecology, Region Västra Götaland, Sahlgrenska University Hospital, Gothenburg, Sweden; 6 Department of Paediatrics, The Queen Silvia Children’s Hospital, Vitaminvägen 21, Gothenburg, 416 85 Sweden

**Keywords:** Oral colostrum, Early colostrum, Colostrum-kit, NICU, Human milk, Oral immunotherapy, Oropharyngeal colostrum, Neonatal nutrition

## Abstract

**Background:**

The World Health Organisation states that newborns should receive colostrum as soon as possible after birth. However, among newborns needing neonatal intensive care, initiation of lactation and access to colostrum might be delayed. At the centre of this study, a tertiary care hospital in Sweden (10,000 deliveries/year), few admitted infants received colostrum within the day of birth, warranting a quality improvement. In order to reduce the time from birth to first colostrum received by infants admitted to the Neonatal Intensive Care Unit (NICU), a new clinical routine including a colostrum-kit, was implemented as standard care in June 2018. The colostrum-kit contained information about hand expression of breastmilk as well as material for collecting, labelling and transporting the colostrum. The kit should be handed to all birthing parents with infants admitted to the NICU.

**Methods:**

Data on time in minutes from birth to first colostrum administered to the infant (oral mouth care, oral feeding or gavage feeding) was retrieved for all infants born between 1 September 2016 and 31 October 2023, admitted to the NICU within 1h from birth. Infants were divided into four time-cohorts, compared with nonparametric ANOVA.

**Results:**

The study included 3618 infants born at 22 + 0 – 43 + 0 weeks gestational age, of whom 2814 (78%) had available data on time to colostrum. Median (IQR) time in hours was 35 (20–36) pre-implementations, followed by 18 (7–38), 11 (4–26) and 8 (3–22) in the subsequent follow-up cohorts, *p* < *0.001*. Subgroups of mode of delivery had median (IQR) pre-implementation of 30 (19–54) for vaginal and 47 (23–72) for caesarean section that reached 7 (2–18) and 9 (3–26) in the last follow-up. Subgroups of gestational age (< 28, 28–31, 32–36, > 36 weeks) had a pre-implementation time of 48 (26–80), 46 (23–73), 33 (20–60) and 32 (19–57), that in the last follow-up was reduced to 4 (2–20), 7 (2–29), 9 (2–33) and 9 (4–19).

**Conclusions:**

Implementing a colostrum-kit for infants admitted to the NICU significantly reduced the time to first colostrum administered to the infant in all gestational ages. The difference between subgroups of gestational age or mode of delivery was reduced. The effect persisted over time.

**Supplementary Information:**

The online version contains supplementary material available at 10.1186/s13006-024-00682-5.

## Background

Human milk (HM) is the golden standard for nutrition of all newborn infants due to its unique properties, not only providing needed nutrients but also being rich in components stimulating growth and development, protecting against infections, and stimulating the immune system [1–3]. Additionally, HM contains cellular components such as neutrophils and macrophages, and a rich microbiota [2]. Enteral feeding with HM to preterm infants almost halves the risk for developing the life-threatening condition necrotizing enterocolitis, compared to feeding with formula [4], and recent studies indicate that feeding with the birthing parents’ unpasteurised own human milk (OHM) is correlated to improved postnatal growth compared to pasteurised donor human milk [5].

The World Health Organization (WHO) recommends breastfeeding within one hour from birth [6] and that preterm and/or sick newborn infants should be given colostrum orally as soon as possible [7]. In reality the access to Own Human Milk for infants in the Neonatal Intensive Care Unit (NICU) is sparse the first days, not only due to delayed lactogenesis II, usually described as the stage of “coming to volume” in milk production [8], but rather often due to lack in lactation support [9, 10].

Unlike the healthy term newborn who in many cases spends the first hours uninterruptedly and independently starting to feed from the breast [11], the preterm or sick newborn is separated from the birthing parent for stabilization and initiation of neonatal care. The start of lactation, normally stimulated by the suckling of the infant, is delayed until other external stimulation occurs, a process that fully depends on a supportive and encouraging environment around the birthing parent with the information, material, and support needed for milk expression [9].

Promoting early initiation of lactation has the potential to result in several positive consequences such as earlier administration of colostrum/OHM to the infant with the potential to reduce infant mortality and morbidity, shorten the time to full enteral nutrition, faster regaining of birthweight, lower incidence of feeding intolerance [12], higher proportion of exclusively breastfed infants at discharge [13, 14], and a lower risk of discontinuation of lactation during the hospital stay [13]. There is a hypothesis of oropharyngeal administration of colostrum promoting a larger immunomodulatory and protective effect than only administering via nasogastric tube [2, 3, 7]. The theory centres on the properties of s-IgA, the main protein in colostrum, which prevents pathogens from penetrating the gastrointestinal mucosa [2]. The effects of oropharyngeal administration of colostrum have not been studied enough to definitively prove its properties in clinical settings [15], but the procedure appears safe for infants of all gestational ages. Further research is performed in countries worldwide.

There are several factors correlated with the provision of own human milk, both in the aspect of early access and in reaching sufficient volumes of OHM through the length of stay at the NICU. Key factors have been described as early breast stimulation (recommendation varies from within 1 h to within 6 h) [16–18], high frequency of milk expression (8–12 times per day) [16], access to hospital grade breast pumps [17], a calming environment, closeness to the infant [9], skin-to-skin care [9, 19], parental information and psychosocial support [9, 10, 17, 19–21].

In this study we hypothesised that by optimising the access to parental information and the material needed for initiating lactation, as well as facilitating the collection and transport of colostrum from the birthing parent to the infant, we could decrease the time from birth to first colostrum received by the infant. The aim of this study was to evaluate the effect of implementing a colostrum-kit on the time to receiving first colostrum, with the primary outcome being time from birth to Time to first Own Colostrum received (TOC) and secondary outcome proportions of infants receiving colostrum within 2, 6, 12, 24, 48 or > 48h respectively (TOC-group), based on timeframes associated with effect of colostrum identified in earlier studies [15, 22–25].

## Methods

### Design and setting

The study was a retrospective observational study, comparing the time from birth to Time to first Own Colostrum (TOC) before and after implementing the colostrum-kit at Sahlgrenska University Hospital in western Sweden. Sahlgrenska University Hospital (approximately 10 000 births/950 NICU admissions/year) is one of six Swedish university hospitals with one NICU level IV, accepting infants from 22 + 0 weeks gestational age (GA), as well as having a NICU level II and neonatal home care [26].

### Intervention

The colostrum-kit was designed by a group of NICU-nurses and midwifes in April 2018. The kit included written parental information in Swedish about initiating lactation and the benefits of colostrum, as well as a 30ml medicine cup with lid, a 1ml syringe, and a sticker for marking the syringe or cup with an identification number, date, and time. The information included a link to a video, showing how to express milk by hand with audio and subtitling in Swedish, published at a website provided by the Swedish healthcare system. Along with the kit a new routine for ensuring early colostrum was implemented. The routine specified that the midwives and assistant nurses at the delivery ward were responsible for supporting the parent in initiating expression of colostrum. Further the routine dictated that the NICU-nurses and assistant nurses were responsible for oral administration of colostrum to the infant as soon as colostrum became available. Education for hospital staff about the routine and the kit was conducted during April 2018 by verbal information during daily meetings and written information sent via email to all healthcare personnel at the delivery wards and NICU. The kit was implemented as standard care on 7 June 2018. The kit was placed in all delivery rooms and in the ward performing caesarean sections, and was handed to the parents by the midwife or delivery assistant nurse in cases of infant admission to the NICU.

### Variables

The primary outcome “postnatal age in minutes when first receiving Own Human Milk” (OHM), in this article referred to as Time to first Own Colostrum (TOC), has been recorded at the clinic as a quality measure since September 2016. The variable includes the administration of colostrum regardless of administration route. Before implementation the administration of colostrum was either orally or by nasogastric tube, depending on the preferences of the nurse. After implementation of the colostrum-kit, the new routine dictated administering colostrum with the syringe orally inside both cheeks of the infant. However, there is no way to identify the mode of administration used for each infant. A large number of variables for evaluating the quality of neonatal healthcare, are both automatically and manually registered in the Swedish Neonatal Quality Register (SNQ) on a daily basis, where the manual registration is conducted by specially trained staff. Eligible infants were identified via the hospital’s SNQ account with a search in the database limited to inborn infants admitted at 0 days postnatal age (PNA) for the specified time-period. In addition to the primary outcome, anthropometric, sociodemographic, and clinical variables were retrieved from the hospital’s SNQ account. Additional review of medical records was done when needed, in order to identify exclusion criteria.

### Participants

Eligible infants were infants inborn at Sahlgrenska University Hospital between 1 September 2016 and 31 October 2023, and admitted to the NICU (either the NICU level IV or the NICU level II) immediately after birth defined as within 1 h postnatal age (PNA). Exclusion criteria were defined as death or decision of palliative care being made within 6 h postnatal age, infants that never received own human milk, infants born outside of the obstetrical delivery ward, infants who needed surgery close after birth, admission at > 1 h PNA, < 24 h length of stay at the NICU, admitted as outpatients, and infants who were breast fed from birth.

### Statistical analyses

To evaluate any effect of the colostrum-kit, participants were divided into four time-cohorts based on date of birth, with a pre-implementation group 0/G0, and three consecutive follow-ups G1-G3, with G0 being born between 1 September 2016 and 7 June 2018, G1 born 8 June 2018 to 29 February 2020, G2 born 1 March 2020 to 31 December 2021, and G3 born 1 January 2022 to 31 October 2023.

Statistical analyses were conducted using IBM SPSS Statistics 28.0.1.1 Central tendencies for time to first own colostrum was calculated both in mean and median, and the choice of analyses was based on the characteristics of the data, using nonparametric median tests with Kruskal–Wallis analysis of variance, due to positive skewness and > 2 cohorts. Categorical variables for TOC-groups, as well as participant characteristics, were analysed using Crosstabulation and Pearson Chi-Square followed by a Tukey HSD Post-Hoc in case of significance. All significance levels were set at 0.05 with a 95% confidence interval. All calculations have been done with Time to first Own Colostrum in minutes but the final results have been converted to hours for easier presentation and interpretation. Gestational age (GA) was defined in completed weeks and sub grouped according to the WHO categories with extreme preterm infants at < 28 weeks GA, very preterm 28–31 weeks GA, moderately preterm at 32–36 weeks GA and term > 36 weeks GA. [27]

## Results

**Fig. 1:**
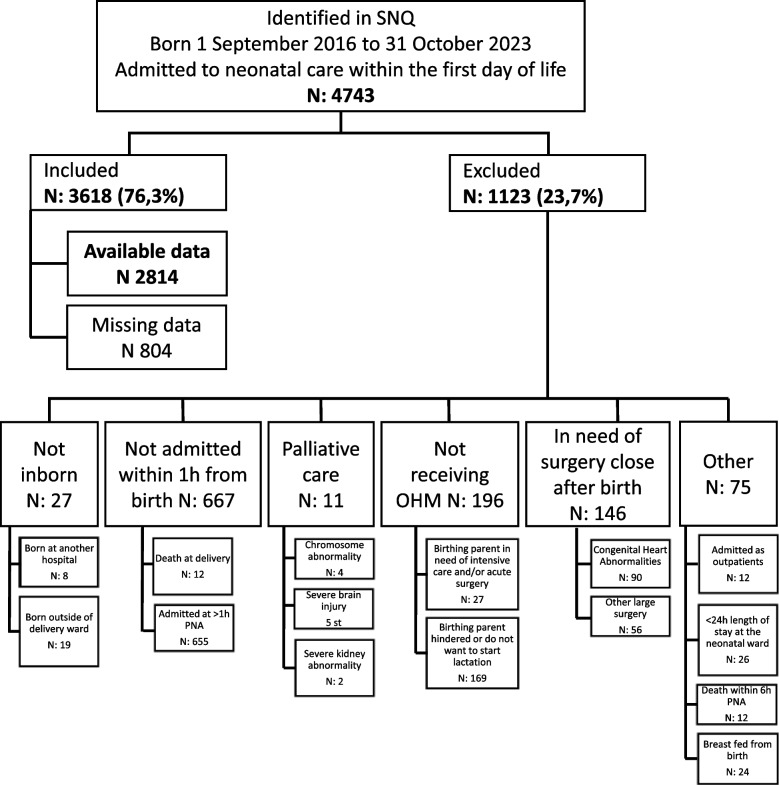
Flowsheet showing the process of identifying participants. Abbreviations: OHM: own human milk; inborn: born at Sahlgrenska University Hospital; h: hour; PNA: Postnatal Age

During 1 September 2018 to 31 October 2023, 4741 infants were admitted to the NICU within the day of birth. Of these, 1123 infants were excluded as shown in Fig. 1, leaving 3618 included infants (3007 were singleton, 581 twins, and 30 triplets from 3060 pregnancies) born by 2336 unique birthing parents (displayed in Additional Fig. 1). Infants included had a mean gestational age at birth of 35 weeks (ranging 22 + 0 – 43 + 0). In 804 (22%) infants, time to colostrum was missing, thus leaving 2814 infants with available data. The infants with missing data had a mean gestational age of 37.6 weeks (Standard Deviation [SD] 3.5) and a mean length of stay of 9 days (median 6), compared to 34.4 weeks (SD 3.8) GA, and mean length of stay 22 days (median 10), in infants with available data (characteristics of infants with missing or available data is presented in Additional Table 1). Out of the 2814 included infants with available data, 632 (22%) were born before implementing the colostrum kit (G0), and in the follow-ups there were 763 (27%) in G1, 814 (29%) in G2 and 605 (22%) in G3. There was no difference between the cohorts in mode of delivery, number of infants with Apgar < 7 at 5 min, or number of infants with antenatal insulin treatment exposure. The proportion of infants in each GA-group had no significant difference between cohorts except for term infants who increased from 34% in G0, to 42% in G3. G3 also had significantly lower proportion of pregnancy/birth-complication than any other group. Group characteristics are presented in Table 1.

**Table 1:** Group Characteristics of the four time-cohorts for the evaluation of implementing a colostrum-kit on time to first own colostrum received

Category	Subgroup	Pre-Intervention G0		Follow-up G1		Follow-up G2		Follow-up G3		TOTAL		Missing	Significance*
		1 Sep 2016 –7 Jun 2018		8 Jun 2018 –29 Feb 2020		1 Mar 2020 –31 Dec 2021		1 Jan 2022 –31 Oct 2023					
		N	valid %	N	valid %	N	valid %	N	valid %	N	valid %		
Gestational Age at birth	<28 w	77	9.0%	111	12.2%	82	8.3%	88	10.1%	358	9.9%	0	No
	28-31 w	119	14.0%	92	10.1%	124	12.6%	87	10.0%	422	11.7%		No
	32-36 w	364	42.8%	364	39.9%	378	38.3%	325	37.5%	1431	39.6%		No
	>36 w	291	34.2%	346	37.9%	403	40.8%	367	42.3%	1407	38.9%		G0 vs G2, G0 vs G3
Mode of delivery	Vag	414	50.2%	480	52.9%	470	47.6%	439	50.6%	1803	50.3%	32 (0.9%)	No
	CS	410	49.8%	428	47.1%	517	52.4%	428	49.4%	1783	49.7%		
Any pregnancy/ birth complications	Yes	214	25.1%	238	26.1%	278	28.2%	172	19.8%	902	24.9%	0	G3 compared to all other groups
	No	637	74.9%	675	73.9%	709	71.8%	695	80.2%	2716	75.1%		
Apgar at 5 minutes <7	Yes	159	19.2%	161	18.1%	158	16.3%	161	18.7%	639	18.0%	71 (2.0%)	No
	No	667	80.8%	729	81.9%	813	83.7%	699	81.3%	2908	80.2%		
Insulin treatment during pregnancy	Yes	27	3.2%	47	5.1%	44	4.5%	30	3.5%	148	4.1%	0	No
	No	825	96.8%	886	94.9%	943	95.5%	837	96.5%	3470	95.9%		
TOC missing		219	25.7%	150	16.4%	173	17.5%	262	30.2%	804	22.2%		
TOTAL included		851	23.5%	913	25.2%	987	27.3%	867	24.0%	3618	100%		

*Abbreviations*: *TOC* Time to Own Colostrum, *w* weeks, *Vag* Vaginal, *CS* Caesarean Section

*Significance at *p*<0.05 within pairwise subgroup comparison using crosstabulation and Pearson Chi-Square

## Central tendencies of Time to first Own Colostrum administered to the infant (TOC)

Median (IQR) TOC decreased subsequently in the four cohorts from initial 35 h (20–62) to 18 h (6.6–38), 11 h (3.5–26) and 8 h (2.7–22) respectively, as viewed in Table 2. The decrease in median Time to first Own Colostrum was significant between all cohorts at *p* = 0.004 for G2:G3 and *p* < 0.001 for all others.

**Table 2:** Time to first Own Colostrum administered to the infant (TOC)

**Category**	**Sub-group**	**G0**	**G1**	**G2**	**G3**	**G0 vs G1 **	**p**	**G0 vs G2 **	**p**	**G0 vs G3 **	**p**
		**Median (IQR)**	**Median (IQR)**	**Median (IQR)**	**Median (IQR)**	**diff**		**diff**		**diff**	
**Full Cohorts**		35 (20-63)	18 (7-38)	11 (4-26)	8 (3-22)	16	<0.001	24	<0.001	27	<0.001
**GA at birth**	<28 w	48 (26-80)	19 (7-55)	9 (3-24)	4 (2-20)	29	0.002	39	<0.001	44	<0.001
	28-31 w	46 (23-73)	19 (5-38)	9 (3-27)	7 (2-29)	28	<0.001	28	<0.001	39	<0.001
	32-36 w	33 (20-60)	19 (6-40)	12 (3-29)	9 (2-22)	14	<0.001	20	<0.001	23	<0.001
	>36 w	32 (19-57)	18 (9-33)	11 (4-24)	9 (4-19)	14	<0.001	21	<0.001	23	<0.001
**Mode of delivery**	Vag	30 (19-54)	13 (5-30)	9 (2-22)	7 (2-18)	17	<0.001	21	<0.001	23	<0.001
	CS	47 (23-72)	24 (9-48)	14 (5-31)	9 (3-26)	23	<0.001	33	<0.001	38	<0.001

Full cohort and subgroup comparison of time in hours from birth to first own colostrum received (TOC), displayed as group median, IQR and difference in median between specified time-cohorts

Time-Cohorts, *G0* pre-implementation, *G1* Follow-up 1, *G2* Follow-up 2, *G3* Follow-up 3

*Abbreviations:* *IQR* Inter-Quartile Range, *diff* median difference, *GA* Gestational Age, *w* weeks, *Vag* vaginal, *CS* Caesarean Section

## Time to first Own Colostrum according to gestational age and mode of delivery

**Fig. 2:**
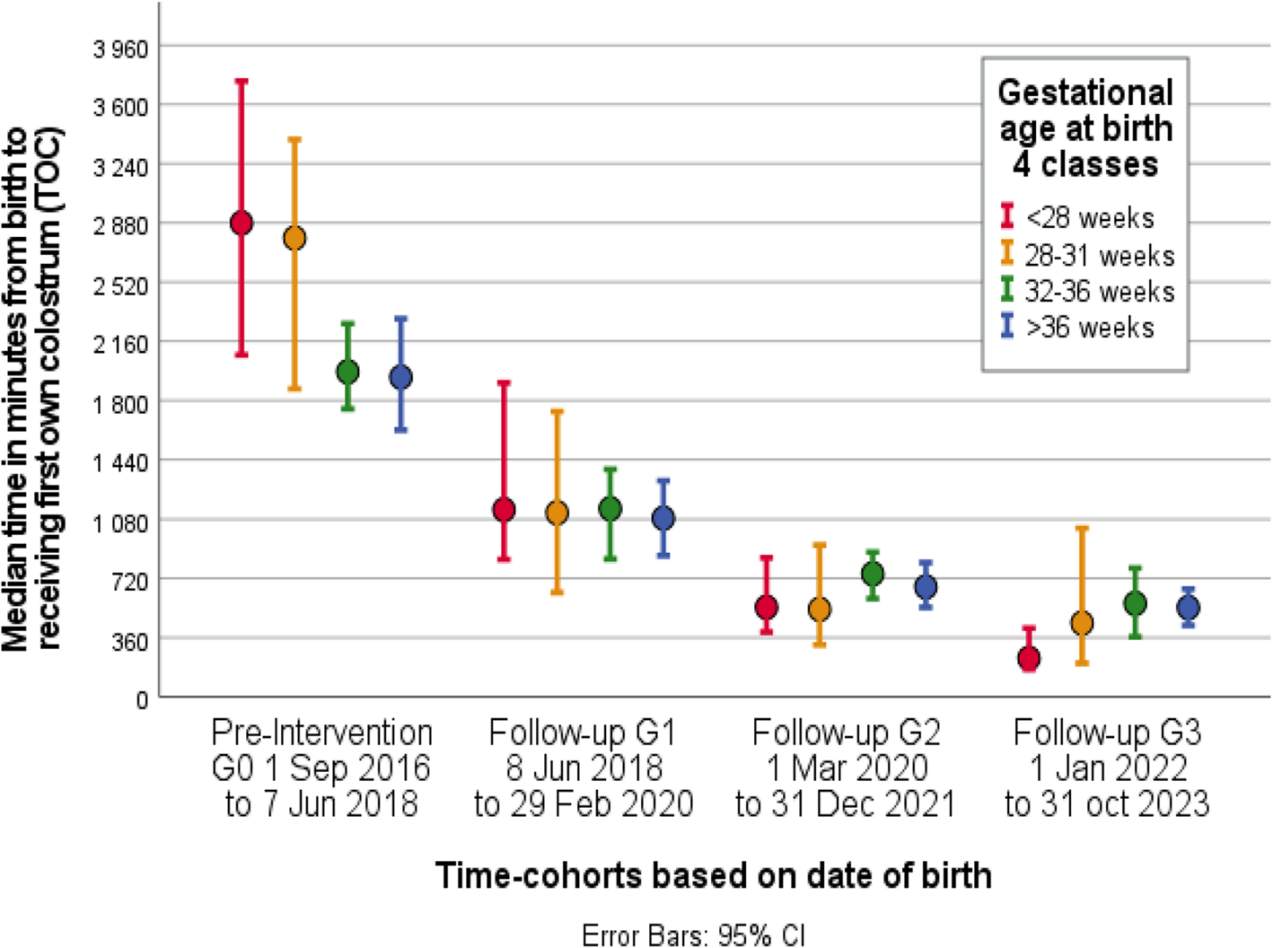
Time to first own Colostrum (TOC) stratified by Gestational Age at birth. Clustered error bar (95% confidence interval), displaying median of time in minutes, from birth to first colostrum received by time-cohorts, stratified by groups of gestational age at birth in completed weeks

**Fig. 3:**
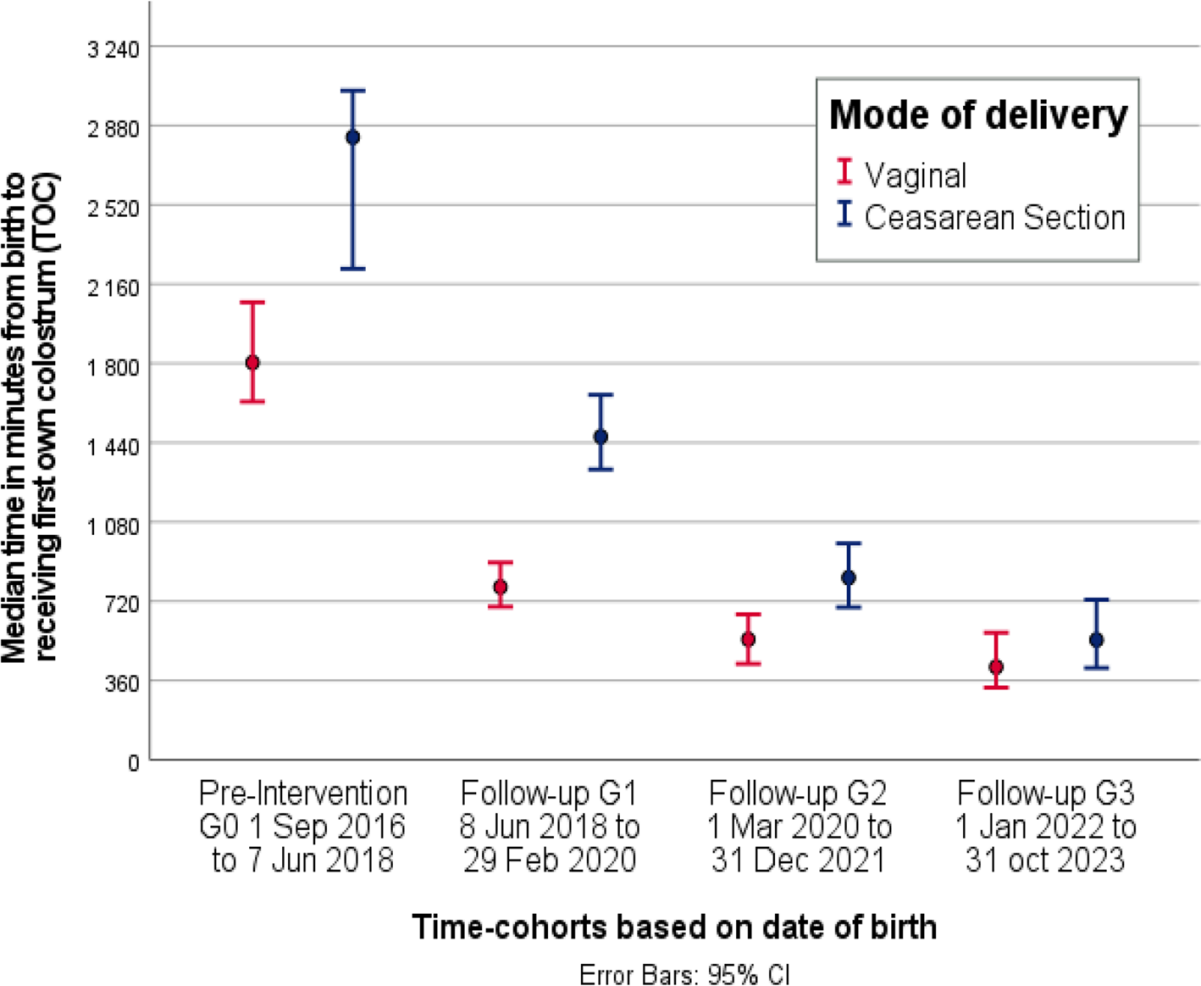
Time to first own Colostrum (TOC) stratified by mode of delivery. Median time in minutes from birth to first own colostrum (TOC) with 95% confidence interval error bars, for each cohort stratified by mode of delivery

When comparing median TOC within subgroups of GA, the decrease was significant in all pairwise comparisons of cohorts for all subgroups, except for G2:G3 within all subgroups, and G1:G2 in GA 28–31 weeks, see Fig. 2. In G0, median TOC differed between groups of mode of delivery, with 30 h for vaginal births and 47 h for caesarean section. There is a significant reduction in median Time to first Own Colostrum in both subgroups and at G3 the difference between the groups is reduced to 7 h for vaginal and 9 h for caesarean section, see Fig. 3.

## Proportions of infants receiving colostrum within different timeframes

**Fig. 4:**
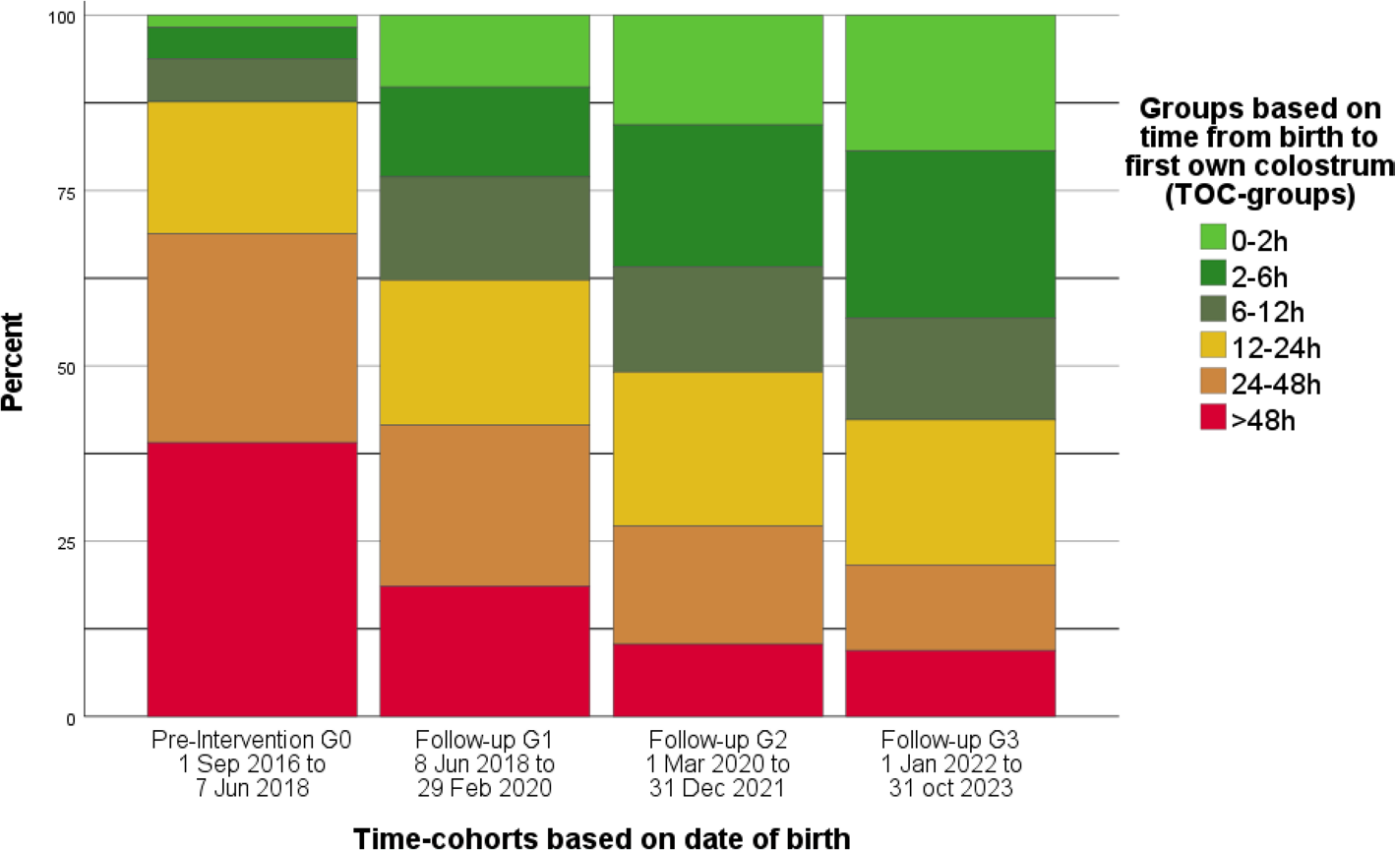
Dispersion of TOC-groups in each time-cohort. Clustered bar chart showing number of infants receiving colostrum within different timeframes in hours from birth (TOC-groups) by time-cohorts

When comparing the proportion of infants in different TOC-groups, there was a significant increase in the groups of 0–2 h and 2–6 h, and a significant decrease in the groups of 24–48 h and > 48 h in relation to the implementation of the colostrum-kit (*p* < 0.001), as shown in Fig. 4 and Additional Table 2. When comparing TOC-groups within the groups of GA the result was similar to the full cohort.

## Discussion

Implementing a colostrum-kit with the information and material needed for collecting the first colostrum from the birthing parent and transport to their infants admitted to the NICU, was significantly correlated with reduced time from birth to first received own colostrum (TOC) with a mean reduction of 29 h. The reduction in TOC was not only rapid and sustained over time after implementing the colostrum-kit, but TOC was subsequently decreasing through the periods of follow-up. Implementing new clinical routines takes time and the effectiveness of the implementation varies with how fast healthcare providers adapt to the new way of working. Therefore, is it not unusual to see a continuous increase in effectiveness after implementation if the belief in the importance of the routine is increasing [28]. The implementation of the kit in clinical care was preceded by information to the clinical staff about the kit and the importance of early initiation of lactation and administration of colostrum. The colostrum-kit serves a practical purpose, increasing the feasibility of early colostrum expression. Beyond its practicality, the routine also highlights the importance of early colostrum administration to the infant. Additionally, the kit was accompanied by a clarification of the healthcare workers responsibilities regarding supporting early colostrum expression. We believe that the continuous decrease in time to first colostrum might reflect an ongoing change in the attitudes of healthcare personnel regarding the importance of early colostrum. Future quality initiatives might supplant or overrun the effect of the colostrum-kit.

Several reports on interventions to increase the availability of own human milk in the NICU have been published, some of multimodal design and others with more isolated interventions. Both lactation rounds, lactation consults, and peer supporters have shown positive effects, as well as kangaroo mother care and early provision of colostrum [19]. Implementing an extensive multimodal quality-improvement programme requires funding and resources for design, implementation and evaluation, and the effect is usually quite limited. The colostrum-kit is on the other hand a low-cost intervention, consisting of material already available and in use, taking an assistant nurse about an hour per week to prepare for a unit with approximately 950 NICU-submissions/year while saving the midwives’ time.

Fleiss et al. performed a quality-improvement study with multimodal design, optimising the knowledge and routines among NICU-staff on early colostrum, increasing the proportion of very low birthweight infants receiving colostrum within 6 h, from 6% (*N* = 70) to 55% (*N* = 37) [22]. When analysing our data in a similar birthweight-cohort results were G0: 6% (*N* = 158), G1: 26% (*N* = 164), G2: 38% (*N* = 163) and G3: 52% (*N* = 151), and thereby reaching the same effect but with probably a more low-cost intervention. In 2018 Kristensen-Cabrera et al. presented a prospective study on a dedicated colostrum collection system for electric breast pumps [29]. The results showed that Primo-Lacto did help facilitate successful colostrum collection. An advantage with the Primo-Lacto is that the system is closed, reducing the risk for contamination, but the cost is higher, and the availability is lower compared to the colostrum kit presented in this study.

### Subgroup effects

The reduction in Time to first Own Colostrum was significant in all subgroups of gestational ages and mode of delivery with a larger reduction in TOC in the group of lowest gestational age and the group delivered by caesarean section. Earlier studies have implied that initiating lactation after very preterm birth and/or caesarean section is complicated due to physiological factors delaying lactogenesis [30]. Murase et al. showed a strong association between caesarean section and delayed initiation of milk expression [14]. Our results showed a large difference in TOC between groups of GA or mode of delivery in the pre-implementation cohort. This disparity corresponds to the belief that parents delivering very preterm or by caesarean section struggles with expressing colostrum. However, after implementation, the difference between these subgroups disappeared. This could indicate that the physiological component for how early after birth it is feasible to express colostrum, is smaller than the component of information and support. It is common for healthcare providers to be cautious about informing and encouraging birthing parents to express colostrum close after giving birth to an infant that needs critical care, even though they are aware of the benefits of own human milk [31]. On the other hand, research shows that families depend on getting early information to be able to make informed decisions about early provision of colostrum, and that the information is effective regardless of socioeconomic background [32]. The nature of the colostrum-kit being easy-accessed and low-effort to distribute to all families promotes a high compliance to the routine, regardless of health staff work-load. The concept of a standardized and clear routine might also bridge the hesitation and cautiousness of many healthcare professionals, making it easier to deliver information in a neutral way, thus increasing the compliance further.

### Implication for clinical care and future studies

There is a need for further studying the effect of early oropharyngeal colostrum since the studies that have been conducted to this date have shown varied results. In the Cochrane report on the subject, the definition of “early colostrum” was set to < 48 h [15], which is quite late regarding the WHO recommendations for immediate expression, and oral administering of colostrum to the sick or preterm infant unable to suckle after birth [7], especially in regard to early nosocomial infections. Our study shows that it is feasible to drastically reduce the time to first colostrum at a low cost, making it possible to conduct studies with narrower definitions on early colostrum that might have an increased chance to produce significant results.

Furthermore, the colostrum-kit has a potential to reduce disparity due to the possibility to have the written information in several languages, with complementing audio–video-instructions and pictograms making the information accessible for different needs and learning-types.

### Strength and limitations

A major strength of this study is the large number of participants with close to 100% participation in the SNQ-register. Confounding variables for the provision of OHM such as GA, mode of delivery and insulin treatment during pregnancy, were available for all participants and comparable between groups. Another strength is the long follow-up of 5 years post implementation showing a persistent reduction in Time to first Own Colostrum.

The single-centre design and a large number of missing observations among term infants are limitations that need to be considered when interpreting the results. However, compared to the other five university hospitals, the study centre had a higher proportion of infants with available TOC data than other centres. In 2022 there were available data for 71% of admitted preterm infants and 39% of term infants at the study centre, compared to 44% and 19% of preterm and term infants respectively admitted to the other five university hospitals combined. The proportion of missing data is higher among term infants, but at the same time the length of stay is significantly shorter among infants with missing data. This could reflect a staff attitude not viewing early colostrum as essential for term infants or when the length of stay is expected to be short. The differences in available data affects the transferability of results regarding term infants with a short length of stay. Additionally, the implemented colostrum-kit only included information in Swedish, limiting the availability for non-Swedish speaking parents.

## Conclusions

Implementing a Colostrum-kit with the information and material needed for early initiation of colostrum expression, is a low-cost and low-effort intervention with a large effect on reduced time to first colostrum received by the infant. Thus, this method is feasible for implementation in both low-income, and high-technological settings, and bridges difficulties transferring information due to language barriers or disabilities.

## Supplementary Information

Additional file 1: Additional Figure 1. Distribution of pregnancies, infants and birthing parents, among included participants. Flowsheet showing properties of included participants.

Additional file 2: Additional Table 1. Comparison of groups with missing or available data on TOC. Table displaying proportions of participants with missing or available data on TOC within different subgroups

Additional file 3: Additional Table 2. Crosstabulation of TOC-groups by time-cohorts. Crosstabulation of groups categorised by time from birth to first colostrum received by the infant, showing variation in proportion between time-cohorts

## Data Availability

The data that support the findings of this study are not openly available due to the General Data Protection Regulation, but can be made available from the corresponding author upon reasonable request. Data can also be retrieved from the Swedish Neonatal Quality Register (https://www.medscinet.com/pnq/). The video on manual milk expression can be found at: https://www.1177.se/Vastra-Gotaland/barn–gravid/att-skota-ett-nyfott-barn/amning-och-flaskmatning/handmjolka-och-pumpa-ur-brostmjolk/ The video on manual milk expression can be found at: https://www.1177.se/Vastra-Gotaland/barn--gravid/att-skota-ett-nyfott-barn/amning-och-flaskmatning/handmjolka-och-pumpa-ur-brostmjolk/

## Abbreviations

GA: Gestational age
HM: Human milk
OHM: Own human milk
NICU: Neonatal intensive care unit
PNA: Postnatal age
TOC: Time to first own colostrum
SNQ: Swedish neonatal quality register

## References

[1] World Health Organization (WHO): Breastfeeding. Available from: https://www.who.int/health-topics/breastfeeding.

[2] Palmeira P, Carneiro-Sampaio M. Immunology of breast milk. Rev Assoc Med Bras (1992). 2016;62(6):584–93. 10.1590/1806-9282.62.06.584. 27849237 10.1590/1806-9282.62.06.584

[3] Embleton ND, Moltu SJ, Lapillonne A, van den Akker CHP, Carnielli V, Fusch C, et al. Enteral nutrition in preterm infants (2022): A position paper from the espghan committee on nutrition and invited experts. J Pediatr Gastr Nutr. 2023;76(2):248–68. 10.1097/Mpg.0000000000003642. 10.1097/MPG.0000000000003642 36705703

[4] Quigley M, Embleton ND, McGuire W. Formula versus donor breast milk for feeding preterm or low birth weight infants. Cochrane Database Syst Rev. 2019;7(7):CD002971. 10.1002/14651858.CD002971.pub5. 31322731 10.1002/14651858.CD002971.pub5 PMC6640412

[5] Lund AM, Domellof M, Pivodic A, Hellstrom A, Stoltz Sjostrom E, Hansen-Pupp I. Mother’s own milk and its relationship to growth and morbidity in a population-based cohort of extremely preterm infants. J Pediatr Gastr Nutr. 2022;74(2):292–300. 10.1097/MPG.0000000000003352. 10.1097/MPG.0000000000003352 PMC8788942 34759238

[6] World Health Organization (WHO). Early initiation of breastfeeding to promote exclusive breastfeeding. Available from: https://www.who.int/tools/elena/interventions/early-breastfeeding.

[7] World Health Organization (WHO), United Nations Children’s Fund (UNICEF). Protecting, promoting and supporting breastfeeding: the baby-friendly hospital initiative for small, sick and preterm newborns. Available from: https://iris.who.int/bitstream/handle/10665/333686/9789240005648-eng.pdf?sequence=1.

[8] Cregan MD, De Mello TR, Kershaw D, McDougall K, Hartmann PE. Initiation of lactation in women after preterm delivery. Acta Obstet Gynecol Scand. 2002;81(9):870–7. 10.1034/j.1600-0412.2002.810913.x. 12225305 10.1034/j.1600-0412.2002.810913.x

[9] Brodsgaard A, Andersen BL, Skaaning D, Petersen M. From expressing human milk to breastfeeding-an essential element in the journey to motherhood of mothers of prematurely born infants. Adv Neonatal Care. 2022;22(6):560–70. 10.1097/ANC.0000000000000962. 34923499 10.1097/ANC.0000000000000962 PMC10519291

[10] Ikonen R, Paavilainen E, Helminen M, Kaunonen M. Preterm infants’ mothers’ initiation and frequency of breast milk expression and exclusive use of mother’s breast milk in neonatal intensive care units. J Clin Nurs. 2018;27(3–4):e551–8. 10.1111/jocn.14093. 28960635 10.1111/jocn.14093

[11] Brimdyr K, Cadwell K, Svensson K, Takahashi Y, Nissen E, Widstrom AM. The nine stages of skin-to-skin: Practical guidelines and insights from four countries. Matern Child Nutr. 2020;16(4): e13042. 10.1111/mcn.13042. 32542966 10.1111/mcn.13042 PMC7507317

[12] Fu ZY, Huang C, Lei L, Chen LC, Wei LJ, Zhou J, et al. The effect of oropharyngeal colostrum administration on the clinical outcomes of premature infants: A meta-analysis. Int J Nurs Stud. 2023;144: 104527. 10.1016/j.ijnurstu.2023.104527. 37295286 10.1016/j.ijnurstu.2023.104527

[13] Raymond M, Gudmundson B, Seshia MM, Helewa M, Alvaro R, Day C, et al. Perinatal factors associated with breastfeeding trends after preterm birth < 29 weeks gestation: Can we predict early discontinuation? J Obstet Gynaecol Ca. 2023;45(1):27–34. 10.1016/j.jogc.2022.11.002. 10.1016/j.jogc.2022.11.002 36436805

[14] Murase M, Nommsen-Rivers L, Morrow AL, Hatsuno M, Mizuno K, Taki M, et al. Predictors of low milk volume among mothers who delivered preterm. J Hum Lact. 2014;30(4):425–35. 10.1177/0890334414543951. 25063573 10.1177/0890334414543951

[15] Nasuf AWA, Ojha S, Dorling J. Oropharyngeal colostrum in preventing mortality and morbidity in preterm infants. Cochrane Database Syst Rev. 2018;9(9):CD011921. 10.1002/14651858.CD011921.pub2. 30191961 10.1002/14651858.CD011921.pub2 PMC6513592

[16] Bendixen MM, Iapicca LC, Parker LA. Nonpharmacologic factors affecting milk production in pump-dependent mothers of critically ill infants: State of the science. Adv Neonatal Care. 2023;23(1):51–63. 10.1097/ANC.0000000000000990. 36700680 10.1097/ANC.0000000000000990 PMC9883598

[17] Meier PP, Johnson TJ, Patel AL, Rossman B. Evidence-based methods that promote human milk feeding of preterm infants: An expert review. Clin Perinatol. 2017;44(1):1–22. 10.1016/j.clp.2016.11.005. 28159199 10.1016/j.clp.2016.11.005 PMC5328421

[18] Parker LA, Sullivan S, Krueger C, Mueller M. Association of timing of initiation of breastmilk expression on milk volume and timing of lactogenesis stage ii among mothers of very low-birth-weight infants. Breastfeed Med. 2015;10(2):84–91. 10.1089/bfm.2014.0089. 25659030 10.1089/bfm.2014.0089 PMC4352698

[19] Hilditch C, Howes A, Dempster N, Keir A. What evidence-based strategies have been shown to improve breastfeeding rates in preterm infants? J Paediatr Child Health. 2019;55(8):907–14. 10.1111/jpc.14551. 31228328 10.1111/jpc.14551

[20] Fleurant E, Schoeny M, Hoban R, Asiodu IV, Riley B, Meier PP, et al. Barriers to human milk feeding at discharge of very-low-birth-weight infants: Maternal goal setting as a key social factor. Breastfeed Med. 2017;12(1):20–7. 10.1089/bfm.2016.0105. 27906557 10.1089/bfm.2016.0105 PMC5220570

[21] Dharel D, Singhal N, Wood C, Cieslak Z, Bacchini F, Shah PS, et al. Rates and determinants of mother’s own milk feeding in infants born very preterm. J Pediatr. 2021;236:21–7. 10.1016/j.jpeds.2021.04.037. 33901519 10.1016/j.jpeds.2021.04.037

[22] Fleiss N, Morrison C, Nascimento A, Stone D, Myers E. Improving early colostrum administration to very low birth weight infants in a level 3 neonatal intensive care unit: A quality improvement initiative. J Pediatr. 2023;260: 113421. 10.1016/j.jpeds.2023.113421. 37076038 10.1016/j.jpeds.2023.113421

[23] Bashir T, Reddy KV, Kiran S, Murki S, Kulkarni D, Dinesh P. Effect of colostrum given within the 12 hours after birth on feeding outcome, morbidity and mortality in very low birth weight infants: A prospective cohort study. Sudan J Paediatr. 2019;19(1):19–24. 10.24911/SJP.106-1540825552. 31384084 10.24911/SJP.106-1540825552 PMC6589796

[24] Pimenta HP, Rocha AD, Guimaraes A, Costa A, Moreira MEL. Oropharyngeal colostrum administration in neonates with gastroschisis: A randomized clinical trial. Crit Care Sci. 2023;35(2):209–16. 10.5935/2965-2774.20230010-en. 37712811 10.5935/2965-2774.20230010-en PMC10406415

[25] Romero-Maldonado S, Soriano-Becerril DM, Garcia-May PK, Reyes-Munoz E, Munoz-Ortiz EG, Carrera-Muinos S et al.: Effect of oropharyngeal administration of colostrum in premature newborns. Front Pediatr 2022;10. 10.3389/fped.2022.891491. 10.3389/fped.2022.891491 PMC9304973 35874579

[26] Papile LA, Baley JE, Benitz W, Cummings J, Carlo WA, Kumar P, et al. Levels of neonatal care. Pediatrics. 2012;130(3):587–97. 10.1542/peds.2012-1999. 22926177 10.1542/peds.2012-1999

[27] World Health Organization (WHO): Who recommendations for care of the preterm or low birth weight infant. https://iris.who.int/bitstream/handle/10665/363697/9789240058262-eng.pdf. 36449655

[28] Penney G, Foy R. Do clinical guidelines enhance safe practice in obstetrics and gynaecology? Best Pract Res Clin Obstet Gynaecol. 2007;21(4):657–73. 10.1016/j.bpobgyn.2007.01.014. 17418642 10.1016/j.bpobgyn.2007.01.014

[29] Kristensen-Cabrera AI, Sherman JP, Lee HC. A prospective clinical study of primo-lacto: A closed system for colostrum collection. PLoS ONE. 2018;13(11): e0206854. 10.1371/journal.pone.0206854. 30418987 10.1371/journal.pone.0206854 PMC6231629

[30] Truchet S, Honvo-Houeto E. Physiology of milk secretion. Best Pract Res Clin Endocrinol Metab. 2017;31(4):367–84. 10.1016/j.beem.2017.10.008. 29221566 10.1016/j.beem.2017.10.008

[31] Sisk PM, Lovelady CA, Dillard RG. Effect of education and lactation support on maternal decision to provide human milk for very-low-birth-weight infants. Adv Exp Med Biol. 2004;554:307–11. 10.1007/978-1-4757-4242-8_28. 15384588 10.1007/978-1-4757-4242-8_28

[32] Meier PP, Engstrom JL, Patel AL, Jegier BJ, Bruns NE. Improving the use of human milk during and after the NICU stay. Clin Perinatol. 2010;37(1):217–45. 10.1016/j.clp.2010.01.013. 20363457 10.1016/j.clp.2010.01.013 PMC2859690

