# Supplementary material for: Implementing a colostrum-kit reduces the time to first colostrum for neonates admitted to the NICU – a retrospective observational study
Source: Int Breastfeed J. 2024 Nov 15;19:77. doi: 10.1186/s13006-024-00682-5 (PMC11566270; doi:10.1186/s13006-024-00682-5)
Supplement: Supplementary file 1 — Additional file 1: Additional Figure 1. Distribution of pregnancies, infants and birthing parents, among included participants. Flowsheet showing properties of included participants. [file 13006_2024_682_MOESM1_ESM.pptx]

## Slide 1
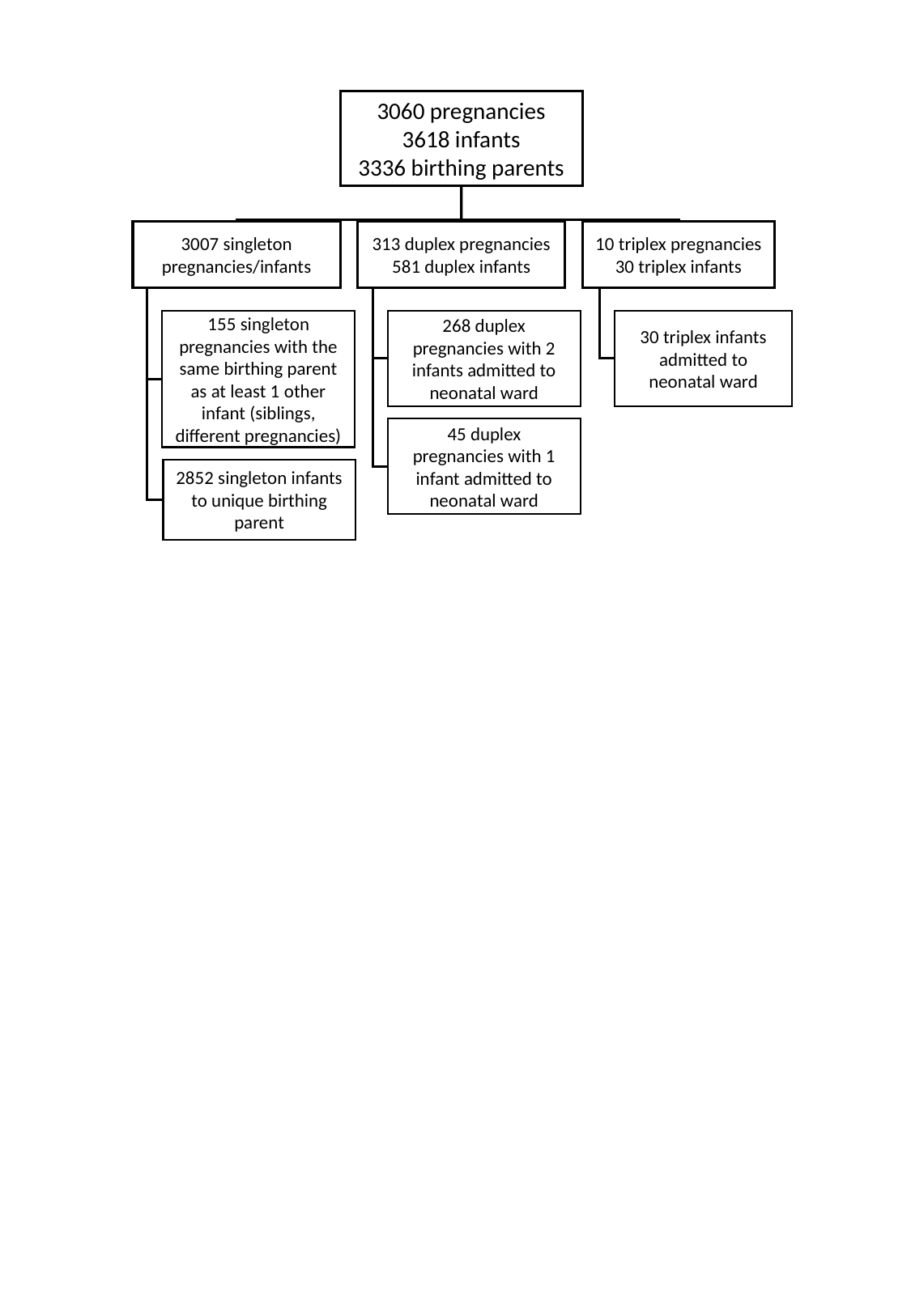

3060 pregnancies
3618 infants
3336 birthing parents
3007 singleton pregnancies/infants
313 duplex pregnancies
581 duplex infants
10 triplex pregnancies
30 triplex infants
155 singleton pregnancies with the same birthing parent as at least 1 other infant (siblings, different pregnancies)
268 duplex pregnancies with 2 infants admitted to neonatal ward
30 triplex infants admitted to neonatal ward
45 duplex pregnancies with 1 infant admitted to neonatal ward
2852 singleton infants to unique birthing parent
